# Predicting the need for a reduced drug dose, at first prescription

**DOI:** 10.1038/s41598-018-33980-0

**Published:** 2018-10-22

**Authors:** Adrien Coulet, Nigam H. Shah, Maxime Wack, Mohammad B. Chawki, Nicolas Jay, Michel Dumontier

**Affiliations:** 1 Université de Lorraine, CNRS, Inria, LORIA, 54000 Nancy, France; 2 Stanford Center for Biomedical Informatics Research, Stanford University, Stanford, California, USA; 3 Service d’Evaluation et d’Information Médicales, University Hospital of Nancy (CHRU), Nancy, France; 4 Institute of Data Science, Maastricht University, Maastricht, Netherlands

## Abstract

Prescribing the right drug with the right dose is a central tenet of precision medicine. We examined the use of patients’ prior Electronic Health Records to predict a reduction in drug dosage. We focus on drugs that interact with the P450 enzyme family, because their dosage is known to be sensitive and variable. We extracted diagnostic codes, conditions reported in clinical notes, and laboratory orders from Stanford’s clinical data warehouse to construct cohorts of patients that either did or did not need a dose change. After feature selection, we trained models to predict the patients who will (or will not) require a dose change after being prescribed one of 34 drugs across 23 drug classes. Overall, we can predict (AUC ≥ 0.70–0.95) a dose reduction for 23 drugs and 22 drug classes. Several of these drugs are associated with clinical guidelines that recommend dose reduction exclusively in the case of adverse reaction. For these cases, a reduction in dosage may be considered as a surrogate for an adverse reaction, which our system could indirectly help predict and prevent. Our study illustrates the role machine learning may take in providing guidance in setting the starting dose for drugs associated with response variability.

## Introduction

Precision medicine aims to improve clinical care using an individual’s information such as genetics, lifestyle, and environment^1^. Considering such information may help in prescribing the right drug at the right dose, and in the process reduce adverse drug reactions (ADR), which are estimated to account for one-third of hospital adverse events and approximately 280,000 hospital admission annually in the US^2,3^. The inter-individual variability in drug responses, including ADR, may have diverse causes such as: *patient conditions*, e.g. a renal dysfunction impacts response to renally excreted drugs; *drug interactions*, e.g. a first drug, such as *fluoxetin* may inhibit the effect of a second drug such as *tamoxifen* by targeting the same enzyme; *drug-food interactions* e.g. aliments such as grapefruit may inhibit drug metabolism enzymes, such as CYP3A4, causing drug toxicity; *genetics* e.g. a genomic variation in the coding sequence of a drug metabolizing enzyme such as CYP3A4 also impacts drug response. The variety of factors, both known and suspected or unknown, makes it challenging for health institutions to take proper precautions^4^.

Electronic Health Records (EHRs) offer novel opportunities for using patient data to study variable patient outcomes including drug response^5^. Research has explored the secondary use of EHRs for predicting disease occurrence^6^, drug effects and their interactions^7^, detecting higher rates of adverse events^8^, and identifying subgroups of drug responses^9^. PheWAS (Phenotype Wide Association Studies) explore the association of a genetic variation with multiple disease phenotypes^10^. Several efforts have associated genotyping from biobanks with clinical data in EHRs^11–13^ and evaluated their potential importance^14^. However, given that genotype data is not yet routinely available in EHRs, an alternative is to use the recorded phenotypes as surrogate markers of individual variations that lead to differential drug response. To the best of our knowledge, no work has yet focused on using EHR data, in absence of genetic information, to predict the variable response to drug exposure.

The goal of this research is to examine the feasibility of using phenotypic data of an individual, recorded in their EHR prior to the drug exposure, to predict a reduced drug-dosing event. Our approach begins with a feature selection step to identify patient characteristics (phenotypes) that are over-represented in patients that needed a drug dose change (increase or reduction) *vs*. those who did not. These selected features comprise *phenotype profiles* that are used for data reduction, prediction and interpretation.

In our work, we considered *drug dose changes* as a sign for individual variation in drug response. In such cases, one may consider the reduction in dosage of a drug as the result of a potential adverse reaction, while an increase in dosage suggests a lack of response. If, however, the dose is unchanged, it suggests an appropriate dosing for the patient.

We focus our study on a set of drugs known to be associated with high inter-individual variability in response, i.e. drugs metabolized by enzymes of the P450 cytochrome family^15^. Genes that code for P450 enzymes have been extensively studied in pharmacogenomics because their variations impact the activity of P450 enzyme and in turn, drug metabolism.

## Results

We examined 34 drugs that are metabolized by P450 enzymes, for which we were able to observe at least 300 drug prescription intervals as described in the methods. We constructed phenotype profiles from patients prescribed one of the 34 drugs by comparing those who experienced a drug dose reduction with those whose dose had not been changed (dose continuation) and by comparing patients who experienced a drug dose increase with those who had a dose continuation. We subsequently evaluated whether phenotype profile were effective in predicting patient drug sensitivity. We found that this method can successfully predict dose reductions for most drugs (23 out of 34), but it could not predict dose increases. We developed a web interface displaying phenotype profiles and the predictions for dose changes for our drugs and their subgroups. This tool was used by three physicians (MW, MC and NJ) to provide clinical interpretation of the reasons why these features may play a role in predicting the need for a lower dose.

### Constructing phenotype profiles for drug dose change

**Figure 1:**
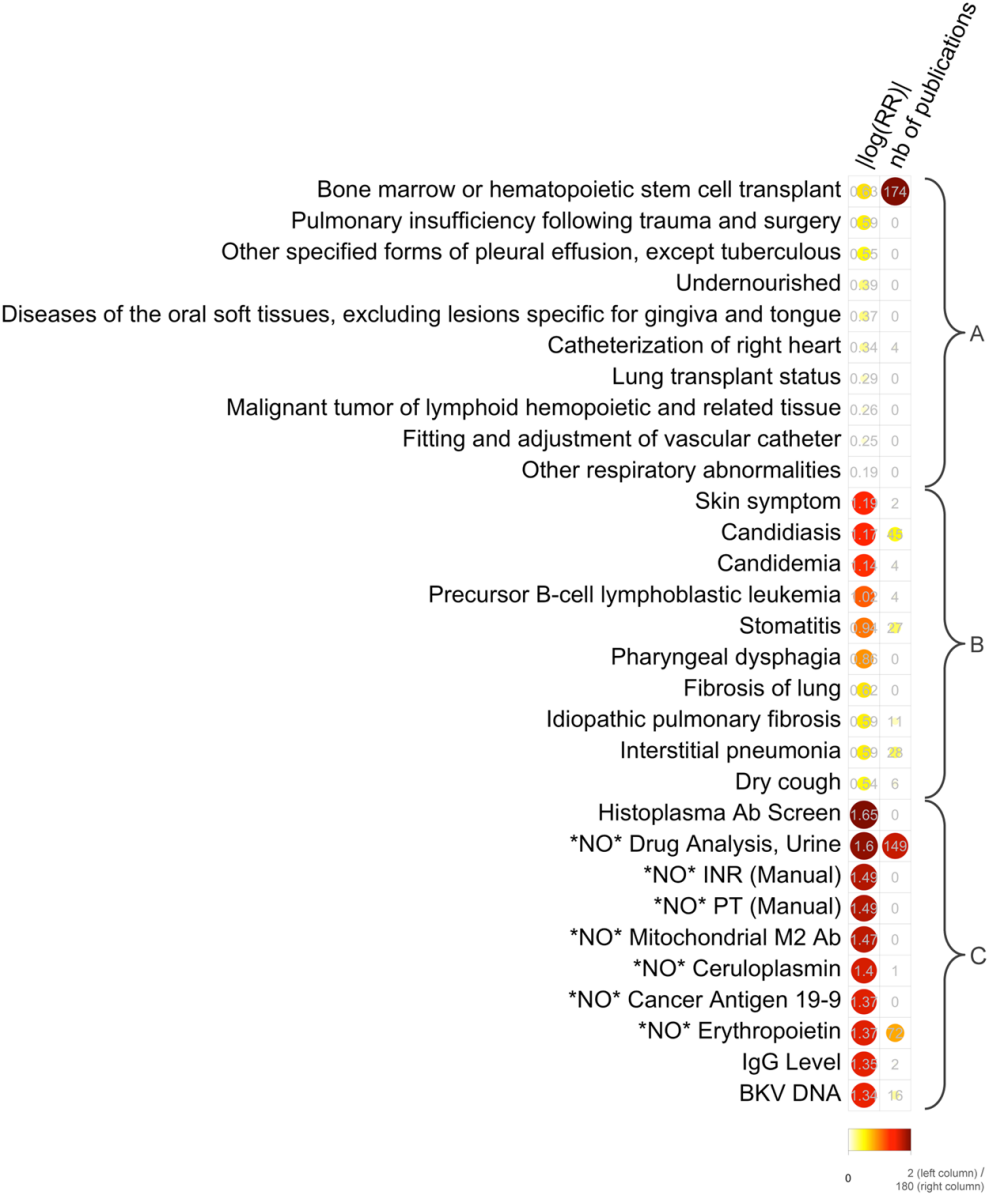
Example of phenotype profile. The shown phenotype profile comprises of the top 10 diagnoses (**A**), condition-mentions (**B**), and lab tests (**C**) that are seen before a tacrolimus prescription in patients who subsequently needed a dose reduction. Only the top 10 in each type are shown. Each phenotype is associated with a statistically significant p-value (hypergeometric test, p < *0*.*05*, Bonferroni correction for multiple testing), and ordered by the absolute value of the log of the Risk Ratio (on a 0 to 2 scale), in the first column. For interpretation purpose, the number of articles in PubMed that mention both the drug and the phenotype is also provided, in the second column (on a 0 to 180 scale). For example, *45* articles mention both *tacrolimus* and *candidiasis*. (**A**) Diagnoses are ICD-9-CM codes associated with patient visits; (**B**) Conditions are phenotypic terms mentioned in text of clinical notes; (**C**) Lab tests are orders of laboratory tests. Lab codes prefixed with the string “*NO*” indicate a negative relationship between the lab test and the drug dose decrease.

Each profile is composed of three *types* of features: diagnostic codes, conditions mentioned in clinical notes and laboratory test orders. Figure 1 shows an example of phenotype profile, containing the top 10 features of each type that are enriched in patients who needed a dose reduction for *tacrolimus*, an immunosuppressant. We generated two types phenotype profiles: the first contains 300 features, composed of the top 100 features of each type (diagnostic codes, conditions, lab orders) and the second is composed of all the features that meet statistical significance. For some drugs, no feature meets with statistical significance, leading to an empty phenotype profile—which means there are no specific characteristics in the patient record that could be indicative of needing a dose change. To allow interpretation of our results, we built a *phenotype profile browser*, available at http://snowflake.loria.fr/p450/. It enables browsing phenotype profiles of drugs and drug sets, and displays the top features of each type. To aid interpretation, phenotype profiles also show the number of publications found in PubMed that mention both the drug and the code/condition/lab test included in the profile.

### Predicting drug dose reduction

We evaluated the effectiveness of phenotype profiles to predict the need for a patient to receive a dose-reduction to a single drug (or to a set of drugs) in two ways: first, by 10-fold cross-validation and second, by holding out the last year of data as a test-set. The first evaluation is a standard setup in machine learning. For the second, the prediction model was trained on patient data from 2008–2013, then tested on the last year of data, i.e., 2014. Drugs and drug sets with less than 300 instances or with an empty phenotype profile were excluded from the evaluation; leaving 34 drugs and 23 drug sets.

Evaluation using 10-fold cross-validation yielded an average AUC-ROC of *0*.*76* and F-measure of *0*.*69*. Average AUC-ROC is *0*.*68*, and F-measure is *0*.*64*, in the hold out evaluation. Results of the dose reduction prediction with an F-measure greater than or equal to 0.7 in the held out evaluation are reported in Table 1. Seven of the top ten results of both evaluations are identical. This difference in discrimination accuracy is partially due to the different sizes of training sets, which is smaller for the second evaluation set up. For single drugs or sets of drugs, both evaluations obtain high values in some cases. For instance, with the 10-fold cross validation we obtain *0*.*94* AUC and *0*.*89* F-measure for tacrolimus; *0*.*93* AUC and *0*.*88* F-measure for the ATC class L-Antineoplastic and Immunomodulating agents. 23 and 10 drugs obtained an AUC ≥ 0.70 with the 10-fold cross validation and the hold out validation, respectively.

**Table 1:** Results of the prediction of dose reduction using phenotype profiles.

Drug or drug set	10-fold cross-validation			Hold last year out		
	|instances|	AUC-ROC	F-m (P; R)	|instances|	AUC-ROC	F-m (P; R)
*Labetalol*	353	0.85	0.77 (0.78; 0.77)	314	0.95	**0**.**86** (0.87; 0.86)
*Tacrolimus*	709	0.94	**0**.**89** (0.89; 0.89)	598	0.94	**0**.**86** (0.86; 0.86)
*Itraconazole*	292	0.88	0.80 (0.80; 0.80)	460	0.86	0.85 (0.88; 0.86)
*Sildenafil*	419	0.88	0.80 (0.81; 0.80)	338	0.90	0.80 (0.80; 0.80)
*Methadone*	941	0.90	0.81 (0.82; 0.81)	866	0.82	0.74 (0.78; 0.75)
*Warfarin*	2851	0.82	0.74 (0.74; 0.74)	2630	0.79	0.72 (0.72; 0.72)
*Hydrocortisone*	4853	0.90	0.83 (0.83; 0.83)	4288	0.80	0.71 (0.72; 0.71)
*H*	17302	0.86	0.78 (0.78; 0.78)	15366	0.76	0.70 (0.70; 0.70)
*2C9*	21607	0.79	0.71 (0.71; 0.71)	19212	0.73	0.70 (0.70; 0.70)
*L*	12119	0.93	0.88 (0.88; 0.88)	10354	—	—
*All P450-drugs*	91267	0.70	0.64 (0.64; 0.64)	80614	—	—

Two evaluations were performed: 10-fold cross-validation and hold last year out. Only the top 100 conditions mentioned in clinical note, top 100 diagnostic codes and top 100 lab codes with a significant p-value (hypergeometric test, p < *0*.*05*, Bonferroni correction for multiple testing) are used in the phenotype profiles. Instances are balanced sets of intervals of dose decrease and dose continuation. Drugs or drug sets associated with a F-measure (F-m) ≥ 0.7 during the hold out evaluation are reported, in addition to results associated with the sets of all P450-drugs and drugs of the ATC class L. L is the first level-ATC classes *Antineoplastic and immunomodulating agents* and H is *Systemic hormonal preparations*, *excluding sex hormones and insulins*. Results for the ATC class L are the best for the 10-fold cross validation, whereas they are not computable for the hold out validation because of empty phenotype profiles in the prediction year. |instances| refers to the number of instances in the training set. Precision (P) and Recall (R) are provided along with the F-measure. Complete results are available in Supplementary material S1. Instance numbers are counted on the train set only and are averaged over the 10 folds of the cross-validation.

Table 2 shows performances using different feature sets for the drug dose reduction prediction. Using all the features does not result in significantly different performance (p-value > 0.05, t-test) than using only 300 features (i.e., the top 100 features of each type). Average F-measures obtained for diagnostic codes, conditions, and lab orders are *0*.*37*, *0*.*4*1 and *0*.*69*, respectively. For most of the drugs and drug sets, lab orders constitutes the most important type of features, while diagnostic codes contributes the least. We are unable to predict dose increases using phenotype profiles (AUC-ROC of *0*.*53* and *0*.*40* for the hold last year out and the 10-fold cross-validation).

**Table 2:** Classifier performance by the type of phenotypic features.

Drug or drug set	F-measure			
	Top 100 diagnostics	Top 100 conditions	Top 100 labs	Top 300 features
*Labetalol*	0.37	0.41	0.81	0.77
*Tacrolimus*	0.41	0.43	0.90	0.89
*Itraconazole*	0.36	0.49	0.79	0.80
*Sildenafil*	0.39	0.42	0.78	0.80
*Methadone*	0.38	0.43	0.82	0.81
*Warfarin*	0.38	0.45	0.76	0.74
*Hydrocortisone*	0.40	0.49	0.82	0.83
*H*	0.37	0.48	0.78	0.78
*2C9*	0.36	0.42	0.71	0.71
*L*	0.41	0.33	0.87	0.88
*All P450-drugs*	0.36	0.45	0.64	0.64

Top 100 lists are ordered on the basis of their p-value. Performances are computed using a 10-fold cross-validation. The top 300 features is the combination of the top 100 features of each type (diagnostic codes, conditions mentioned in clinical notes and lab orders).

### Interpretation

We reviewed prescription guidelines and sections *“dosage and administration”* of drug labels for the 23 drugs for which we were able to predict dose reduction to verify if dose reductions were recommended in the case of an undesirable response, or if it may be part of a regular protocol of drug prescription. For 14 drugs, out of 23, a dose reduction is only recommended in the case of an undesirable response; For the other 8 drugs, a dose reduction is recommended either in the case of an undesirable response or in normal prescription management. In our top-prediction list provided Table 2, dose reduction is recommended only in the case of an undesirable response in 5 out of 7 drug guidelines; dose reduction is part of the normal prescription for the other 2 drugs (hydrocortisone and methadone). Lists of drugs and their guidelines are provided in Supplementary file S1.

We examined phenotype profiles of four drugs (labetalol, tacrolimus, warfarin and sildenafil) to understand why phenotype profiles enable the prediction of dose reductions for those drugs before their first prescription. Phenotype profiles reviewed are in Fig. 1 and in Supplementary Fig. S2.

The profile of tacrolimus, an immunosuppressant used in patients with organ transplants, shows that dose reduction intervals for this drug (in comparison with drug continuation intervals) is negatively associated with previous urine drug analysis (see Fig. 1). We also found that on average there were more intervals of dose reduction per patient for this drug than for others (i.e., 3.13 dose reductions per patient for tacrolimus and 1.44 (SD = 0.40) dose reductions on average for all drugs). This, and association with urine drug analysis, can be explained by the fact that tacrolimus dosage is often re-evaluated to get to the optimal dosage, with the help of urine drug analysis. The negative association with urine analysis is expected due to more dose continuation following a normal analysis.

The profile of labetalol, an alpha/beta adrenergic antagonist use to treat essential hypertension, shows that its dose reduction is strongly and positively associated with prerenal renal failure and acute renal failure diagnostics as well as lab tests for renin activity and aldosterone (see Supplementary Fig. S2). These diagnostics are associated with non-essential hypertension, and can explain a dose reduction of the first intention treatment, after specific tests for secondary hypertension. Renin activity and aldosterone testing are indeed used to diagnose secondary causes of hypertension.

Warfarin is an anti-vitamin K anticoagulant used to prevent the formation of blood clots. Interestingly, dose reduction is associated with candidiasis (see Supplementary Fig. S2), a diagnosis that leads to prescription of anti-fungal drugs such as miconazole, which are contra-indicated in conjunction with warfarin, because of their inhibitory interaction with cytochrome P450, of which warfarin is a substrate. Warfarin dose reduction is also associated with chronic renal failure, a contra-indication for warfarin prescription, as well as with clot kinetics lab tests, standard control tests for dose adaptation of anticoagulant drugs. We did not find association with INR (International Normalized Ratio) testing, probably because INR is monitored during dose adjustment as well as routinely after a stable therapeutic dose is found.

Lastly, sildenafil is a phosphodiesterase inhibitor used in erectile dysfunction and pulmonary arterial hypertension. Sildenafil profile show that its dose reduction is associated with the presence of thromboembolus, a major contraindication for sildenafil prescription, as well as with bilateral pleural effusion, a symptom of cardiac congestion, and another contraindication for sildenafil prescription (see Fig. S2). Additionally, we found that the sex ratio in the patients who had a dose reduction was 1:1 for men and women, whereas the sex ratio for dose continuation was closer to three men for one woman, indicating that dose reductions mostly happened in patients for whom sildenafil was prescribed for pulmonary arterial hypertension.

## Discussion

We trained classifiers to predict dose changes using three types of features from EHR data. Our underlying assumption is that the need for dose changes is marker for an inappropriate drug response, such as a drug adverse response or lack of response. Our classifier (trained on the phenotypic profiles) successfully predicts dose reductions for 23 drugs out of 34 drugs in the study, but is ineffective in predicting dose increases. We also found that the laboratory test orders generated better predictions than other features. Finally, we find that when evaluating the prediction ability using a held out test set from a later calendar year (i.e., totally unseen ‘future data’), the performance is lower than, but consistent with, the commonly done 10-fold cross validation.

For predictive models in medicine, an unseen *future* dataset is the most appropriate evaluation setup because it mirrors real-life usage of a model. Even in this stringent setting, we are able to predict dose reductions for 10 of the 29 drugs. Note, that in this prospective prediction setup, not all of the 34 drugs in the study have enough training and test data to build models.

Our work, while offering a proof of the ability to use EHR data for predicting the need for a future drug dose reductions, has several limitations. Dosing changes is part of regular prescription protocol of some drugs and in this case a prediction does not inform patient care. In our study glucocorticoids, such as hydrocortisone and dexamethasone, have guidelines that recommend a dose reduction independently of the drug response. However, for most of the drugs in question (14 out of 23), prescription guidelines recommend dose reduction only in the case of an unwanted reaction. Some drugs we studied (e.g., tacrolimus, warfarin) have guidelines recommending for dose adjustment (up or down). In this case, our system may help by predicting the need for a dose reduction, before first prescription, potentially reducing the necessary time to reach stable dose for these treatments. Another limitation is that we do not account for cases in which adverse drug responses are managed by drug discontinuation or exchanging one drug for another. Accounting for drug discontinuation and switching may improve our performance. We neither consider the co-administration of multiple drugs. Drug prescribed at prediction time, or before, could also be considered as additional feature.

While, we are able to predict dose reductions, the approach fails in predicting dose increases. Our analysis revealed that there are very few conditions mentioned in clinical notes associated with a dose increase for P450-drugs. This lack of observable features for the “absence of a drug response” can be due to lack of reporting in the EHR or to the inability to identify these from EHR. Even for drugs or drug classes associated with good prediction metrics, we observed some false positive and false negative. Those cases are hard to interpret because first of the dimensionality of the model, second because the encoding of the data loose the temporal order of features, making impossible, from this encoding to retrieve the history of a patient. It seems however and unsurprisingly that our model mistakes with patients associated with a lighter density of features.

A third limitation is that while the order of a laboratory test was an important contributor to the performance of the classifier, we have not explored the use of test result values. Result values may enable detection of lack of response to drugs and enable predicting dose increases. Finally, we used a hypergeometric testing with the Bonferroni correction, which is highly stringent, whereas the Holm-Bonferroni^16^ is more sensitive.

While our results are promising, more must yet be done to improve the approach. Next generation learning algorithms such as recurrent neural networks may provide substantial performance gains. Considering additional confounding factors may also increase the global performance. Manual chart review or linkage to a biobank with genotype data for the identification of a set of patients with known drug sensitivity could provide further clinical validation. One challenge is also to combine our data-driven approach, with approaches based on broad mechanistic understanding and knowledge of the human physiology as evoked in^17,18^.

In conclusion, we demonstrate that the phenotypic history of a patient available in the EHR, may be used in machine learning algorithms to identify patients who will likely need a drug dose reduction. Future efforts can focus on a combined strategy for guiding personalized drug prescription, including genetic testing and prior phenotypic data. Several projects such as the Vanderbuilt PREDICT, the Mayo Clinic RIGHT programs successfully demonstrate how the genetic determinants in drug response variability may be beneficially used to individualize drug prescriptions and reduce ADR^19–21^. However, for many situations, the genetic determinants are useful, but not sufficient and may benefit from additional, phenotypic features to guide drug prescription. In this particular case, machine learning approaches such as the one presented in this paper would complement pharmacogenetics testing^22^. The opportunity is underscored by the fact that roughly 10,620,000 individuals per year in the US receive a new prescription for one of the 34 drugs we built models for (estimated using 2007–2014 Truven MarketScan Commercial Claims and Encounter database). The ability to prospectively identify a large fraction of these individuals (roughly 1,949,000 per year for the 10 drugs for which the dose reduction prediction works well) is highly significant.

## Methods

**Figure 2:**
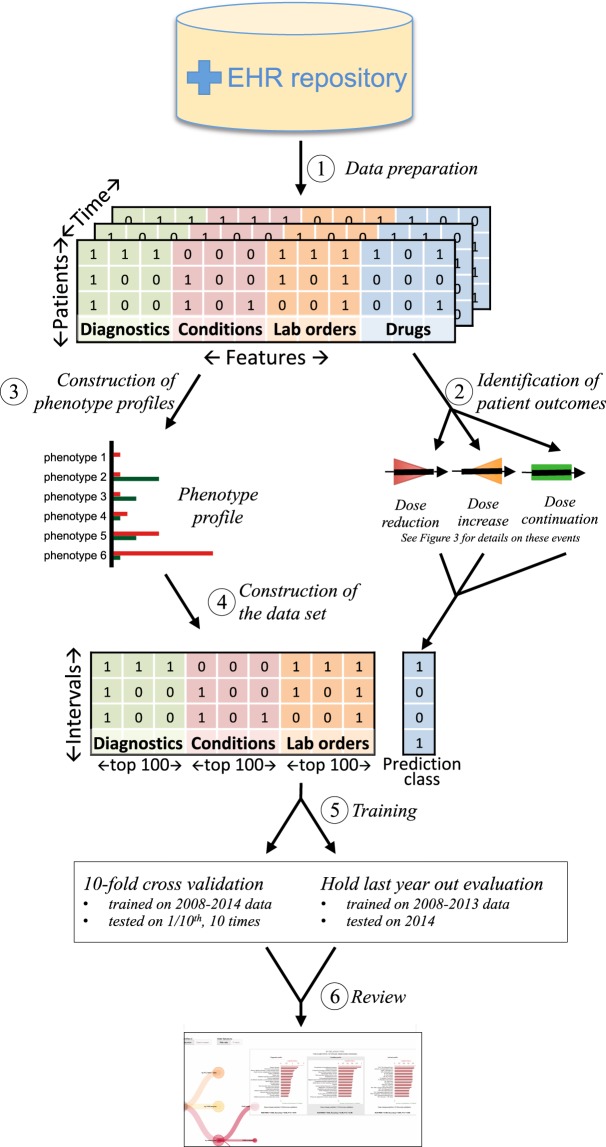
Overview of our approach of predicting the need for a reduced drug dose, at first prescription. (1) Clinical conditions mentioned in clinical notes are identified using terms from medical ontologies; other phenotypic features encoded in Electronic Health Records are directly extracted. (2) Drug dose changes and continuations are detected. (3) Characteristics associated with dose changes are identified to construct phenotype profiles. (4) Phenotype profiles are used to filter the features and build a reduced data set. (5) The resulting matrix is then used to train two binary random forest models: one for predicting dose reductions and one for dose increases. (6) We used two evaluation setups and review by experts to determine the performance of the models.

An overview of our approach is presented in Fig. 2. The following section details each step and describes the drugs we consider in this study. The work was done after IRB approval (#24883) at Stanford University, with informed consent for study participation.

### Drugs analyzed

We considered in this study drugs whose metabolism is impacted by enzymes of the family of P450 cytochrome, known to interact with many drugs and xenobiotics. We refer to those as *P450-drugs*. Flockhart proposed a list of P450-drugs^23^ that is manually reviewed by experts of P450 enzymes and provides elements of evidences in the form of PubMed references. Because there may be too few patients corresponding to a single P450-drug in our data, we also perform the analysis for ‘sets of drugs’. Following the list of Flockhart and the ATC (Anatomical Therapeutic Chemical) classification of drugs^24^, we identified 25 sets of P450-drugs grouped accordingly to three distinct criteria. To minimize bias related to frequently prescribed drugs we excluded the drugs that were prescribed more than *55*,*000* times. Of the 205 drugs grouped into 25 drugs sets, we also excluded those associated with too few training data, using an arbitrary threshold of 300 drug intervals (≥150 dose reductions/increases and ≥150 dose continuations). Only 34 drugs across 23 drug sets had enough. Drug sets and their size are summarized in Supplementary Fig. S3, and made available in JSON format in Supplementary file S4.

### Dose change and dose continuation intervals

We use EHRs from the STRIDE clinical data warehouse^25^. It comprises of *1*,2*50*,*8*2*5* patients who visited Stanford Hospital and Clinics between 2008 and 2014, constituting *49*,*086*,*060* visits, *27*,*049*,*309* clinical notes, *19*,*435*,*069* drug orders (including *2*,*891*,*470* for P450-drugs) and *165*,*141*,*675* laboratory test orders. We defined “dose change intervals” as follows: *Dose reductions* are temporal intervals during which a patient was twice prescribed the same drug ingredient with a decreased dose on the second prescription, within 20 days, using the same route of administration and reported with the same unit; *Dose increases* are temporal intervals in which a patient was twice prescribed the same drug ingredient with an increased dose on the second prescription, within 20 days, using the same route of administration and reported with the same unit; Finally, *dose continuations* are intervals between two prescriptions in which the dose, the route and the unit are unchanged. The arbitrary chosen length of 20 days for intervals is supported in our study of P450-drugs by the relatively short length of intervals we observed in EHR (3.64 days, standard deviation = 4.41).

**Figure 3:**
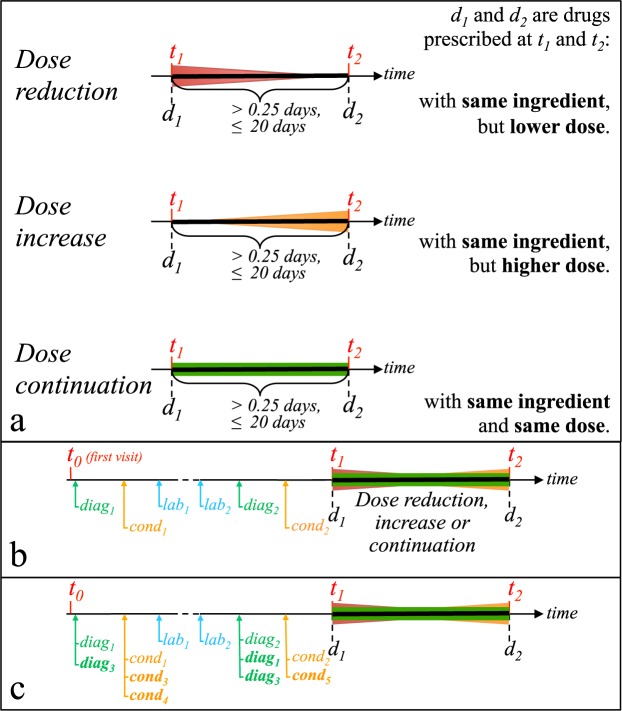
Definition of the dose change intervals and construction of features. The top panel (a) Shows three kinds of intervals. Each interval is delimited by two drug prescriptions *d*_1_ and *d*_2_, prescribed at *t*_1_ and *t*_2_. *d*_1_ and *d*_2_ have a same ingredient. No other drug *d*_*i*_ with the same ingredient is prescribed between *t*_1_ and *t*_2_. The bottom panels show the construction of phenotypic features and their expansion using ontology hierarchies. (**b**) The three kinds of features are: diagnostic codes (*diag*_*i*_), conditions mentioned in clinical notes (*p*_*j*_) and laboratory test orders (*lab*_*k*_). Diagnostic codes and test orders are available in EHRs, whereas condition mentions result from automated annotation of clinical notes. (**c**) The diagnostic codes and conditions found in clinical notes are generalized according to ICD-9-CM and SNOMED-CT, respectively. For example, if ICD-9-CM states that *diag*_3_ is more general than *diag*_1_, then *diag*_3_ is also associated with the interval. Generalization is not done for lab tests because too few of them are mapped to an ontology. All features are constructed from EHR data before the first prescription (*d*_1_) of intervals.

Dose reductions are either a decrease in the quantity of drug prescribed or a decrease of the frequency of prescription. Similarly, dose increases are either an increase of quantity or a increase of frequency. Figure 3 summarizes the three kinds of intervals. We eliminated outlier intervals by excluding the 10% intervals either too short (<6 hours) or too long. In addition, we consider as ‘dose continuations’, only intervals of patients who never experienced a dose change (up or down). However, ‘dose changes’ we consider might precede or follow some dose continuation intervals. We identified *50*,*704* dose reductions, *60*,*719* increases and *176*,*140* continuations in the prescriptions of P450-drugs in STRIDE.

### Features observed before dose changes and continuations

We considered three distinct types of features, each observed before the first prescription of the interval (i.e., *d*_*1*_ in Fig. 3): diagnostic codes, conditions mentioned in clinical notes and laboratory orders.

*Diagnostics codes* are encoded with the ICD-9-CM (International Classification of Disease, Ninth Revision, Clinical Modification) in EHRs^26^. ICD-9-CM codes are associated with each visit of a patient at the hospital, documenting the main reasons for the admission, and the main events that occurred during the stay of the patient, reported at discharge.

*Conditions mentioned in clinical notes* are disease and symptom terms mentioned in clinical narrative notes. They are obtained by processing the clinical notes, using a pipeline described in^8^. This pipeline ignores the mentions of concepts that are negated and those that are mentioned in the patient family history. Its performance for event identification, reported in^8^, are of 74% sensitivity and 96% specificity, but accuracy varies by condition. In this work, we only keep annotations made with concepts that are part of the SNOMED CT^27^. For the selection of SNOMED CT conditions only, we manually defined a subset of UMLS semantic types^28^ to consider from: ‘Disease or Syndrome’, ‘Mental or Behavioral Dysfunction’, ‘Cell or Molecular Dysfunction’, ‘Event’, ‘Sign or Symptom’, ‘Anatomical Abnormality’, ‘Neoplastic Process’.

*Laboratory test orders* are structured data that indicate that a laboratory test, such as *O*_*2*_ *saturation*, has been ordered for a patient. Unfortunately, our view of the EHR data contains sparse lab results, whereas all orders are listed. While lab orders are not exactly phenotypic observations, they can be considered proxies for a suspected underlying condition. Only 2 in 10 lab orders were encoded with a terminology in our dataset, therefore we used the laboratory order codes directly rather than mappings to a reference terminology.

We used the hierarchies of ontologies to generate an expanded feature set, as illustrated in Fig. 3. If a dose reduction interval is associated with the SNOMED CT concept *Stomatitis*, then it will automatically be associated with parent terms *Inflammatory disorder of digestive tract* and *Disorder of digestive tract* as defined in the SNOMED CT hierarchy. This expansion, on the parent-child hierarchy of the ontology, aims at capturing more information and improving classification of dose changes. Diagnostic codes and conditions are expanded using ICD-9-CM and SNOMED CT respectively, whereas lab orders were not associated with any hierarchy, thus not expanded. Table 3 reports the number of phenotypic features prior and following ontology expansion.

**Table 3:** Numbers of intervals of each type and number of their associated phenotypic features.

		Dose reduction	Dose increase	Dose continuation	Total
*Intervals*		50,704	60,719	176,140	287,563
*Patients*		22,571	25,381	56,902	69,308
*Diagnostic codes*	*before expansion*	1,434,606	1,623,309	4,088,965	7,146,880
	*after expansion*	3,687,022	4,193,204	10,811,286	18,691,512
*Conditions from clinical note*	*before expansion*	441,746	505,235	1,173,403	2,120,384
	*after expansion*	4,110,928	4,728,006	11,487,221	20,326,155
*Lab test orders*		6,773,097	7,906,040	17,896,716	32,575,853

Diagnostic codes and condition mentions found in clinical notes are expanded using ICD-9-CM and SNOMED CT, respectively.

### Constructing Phenotype Profiles

We adopt the approach proposed by Lependu *et al*.^29^ that applied enrichment analyses^30^ to disease and phenotype studies. Gene expression profiles are commonly computed in transcriptomics to study how gene expression varies depending on conditions such as diseases or treatments. A “profile” in this case is a set of genes differentially (i.e., over- or under-) expressed in one condition compared to another, such as the presence *vs*. the absence of a disease. Accordingly, gene expression measurements may be replaced by diagnostic codes, condition mentions, or lab test orders in patient EHRs, and the enrichment analysis highlights codes, conditions, or lab tests that are over-represented in a group of patient compared to another.

In this study, we construct phenotype profiles from patients prescribed specific drugs by comparing those who experienced a drug dose reduction with those who had a dose continuation; and comparing patients who experienced a drug dose increase with those who had a dose continuation.

The over-representation of a specific *feature*, is quantified by its *p-value*, computed using the hypergeometric test. A p-value quantifies how likely is it that a feature is associated with a dose reduction (or increase) by random chance. We also compute *Risk Ratio* (RR) and *Information Content* (IC) for each feature. The RR measures the strength of association of a feature with the with dose reduction (or increase) of a drug. The IC is used to exclude features that may be either too common or too uncommon in our set of EHRs. Details on the computation of p-value, RR and IC are provided in supplementary methods.

Phenotype profiles are initially composed of features with a p-value < *0*.*05* (hypergeometric test). Then, first we remove features with a 0.5 < RR < *2*. Second, features with an IC either in the first or the fourth quartile are excluded. For condition mentions in clinical notes, this step consists of keeping only conditions with *4*.*25* < IC < *12*.*75*. Third, only statistically significant associations, after correction for multiple testing are kept (p < *0*.*05*, hypergeometric test, Bonferroni correction). Fourth, we applied the *elim* method described by Alexa *et al*.^31^ to deal with features added due to the ontology expansion. One issue with this process is that general concepts from the ontology tend to dominate the set of results. The *elim* method avoids this drawback by eliminating every feature that is more general than another, but is associated with the outcome (dose reduction, increase or continuation) with an equal or higher p-value. Table 4 reports numbers of features remaining after each filtering step. The order of the four steps has been chosen for processing efficiency, but could be executed in any order. The resulting phenotype profiles are used to construct train and test sets, which in turn are used to learn and evaluate our predictive model. The numbers of publications that come along phenotype profiles are obtained using E-utilities, an API provided by the National Center for Biotechnology Information^32^. The query for publication numbers is built as the conjunction of two labels: the label associated with the drug (or drug class, see Supplement Material S4 for labels) and the preferred label associated with the feature, following ICD-9 for diagnostics, SNOMED CT for conditions or STRIDE for labs.

**Table 4:** Reduction of the size of phenotype profiles.

	Dose reductions			Dose increases		
	diagnostics	conditions	labs	diagnostics	conditions	labs
*Total number of features*	213,737	92,255	106,041	67,722	23,539	24,231
* after RR filtering*	104,414	42,225	50,572	41,796	15,122	14,961
* after IC filtering*	88,973	13,552	34,277	29,875	5,353	5,974
* after p-value correction*	31,218	3,553	24,442	27,071	5,353	5,974
* after elim method*	18,256	1,947	—	11,838	1,932	—

This table reports the number of features of each type that comprise phenotype profiles at various steps of filtering. RR and IC filtering steps are based on Risk Ratio (RR) and Information Content (IC). The *elim* method helps filtering results of the ontology expansion process^31^. This method cannot be applied to laboratory test orders since those are not encoded with ontology codes.

### Training and evaluation of the predictive model

We trained two binary classifiers using the Random Forest algorithm^33^ to predict dose reduction or dose increase as compared to dose continuation. First, phenotype profiles are pruned by selecting features as described before, prior to training. For each classifier, and for each drug and drug set considered, we filtered out two sets of phenotype profiles: a set of profiles with 300 features, composed of the top 100 features of each type (diagnostic code, conditions, lab orders) and a set with profiles composed of all the features that meet statistical significance.

The training sets are balanced, i.e., they include the same number of dose reduction (or increase) intervals as continuations. Features present in the history of patients were encoded with 1, whereas absent or missing features were encoded with 0. Drugs and drug sets with less than 300 instances or with an empty phenotype profile (i.e., no feature was found with sufficient p-value, RR and IC) were excluded from the evaluation; leaving 34 drugs and 23 drug sets to build classifiers for. Every classifier is evaluated with a *10-fold cross-validation* and *hold last year out* validation. In each evaluation setting, the feature selection was achieved on the training data only, meaning that a distinct phenotype profile was computed for each fold of the 10-fold cross-validation. In the hold last year out setting, 2014 data that are left out comprises respectively *17*.*5*%, *18*.*0*% and *18*.*9*% of the dose reduction, increase and continuation intervals of the 2008–2014 data set. Such hold out evaluation illustrates the ability of our model to perform prediction on totally unseen data and also provides the most conservative estimate of performance in the face of data non-stationarity^34^. Computation of the Random Forest is achieved with the Weka 3.8 toolbox^35^, with 100 estimators, an unlimited depth of trees, and *int*(*log*_2_ (*|features|*) + *1*) as the number of features considered at each split.

### Assessing the association between dose decrease and inappropriate drug response

We manually reviewed prescription drug labels from the DailyMed website (https://dailymed.nlm.nih.gov) and when available additional prescription guidelines. From this review we established if it was recommended adjusting the dose of the drug only in the case of inappropriate responses, or if a dose change may be recommended in a normal prescription settings of the drug. Results of the review and references used are provided in Supplement Material S1.

In addition to this review, we counted from clinical notes and for each drug, the number of mentions of conditions classified as Adverse Drug Responses (ADR) according to^36^, during intervals of time of dose reductions vs. dose continuations, as defined in Fig. 3. We used these counts to compute the *Risk Ratio*, which provides an estimate of the disproportion for ADR to occur during dose reduction intervals in contrast with continuation intervals. Results form this evaluation are reported in Supplementary result S1.

### Interpretation of predictions

For interpreting the relationships between features in phenotype profiles and drug sensitivity patterns observed, we (MW, MC, and NJ) reviewed the results and investigated the biomedical literature. To facilitate our interpretation, we developed a web application with the JavaScript library D3JS (https://d3js.org), available at http://snowflake.loria.fr/p450/.

### Ethical approval and informed consent

The work was done with IRB approval (#24883) at Stanford University.

## Electronic supplementary material

Supplementary Information

Data File S1

Data File S4

## Data Availability

The research uses de-identified EHR data from the Stanford University School of Medicine’s clinical data warehouse. Given the regulations governing the access of patient data, the data can not be deposited in a public repository. All other data mentioned in this manuscript are available online as supplementary information. Programmatic code used in this work is available at https://github.com/coulet/PredictDoseReduction.
