# Supplementary material for: Predicting the need for a reduced drug dose, at first prescription
Source: Sci Rep. 2018 Oct 22;8:15558. doi: 10.1038/s41598-018-33980-0 (PMC6197198; doi:10.1038/s41598-018-33980-0)
Supplement: Supplementary file 1 — Supplementary Information [file 41598_2018_33980_MOESM1_ESM.pdf]

Supplementary Information

Manuscript title: Predicting the need for a reduced drug dose, at first prescription

Authors: A. Coulet, N. H. Shah, M. Wack, M. B. Chawki, N. Jay, M. Dumontier

Data File S1. Complete results of the evaluation of the drug sensitivity prediction.
This XLSX file lists the results of the evaluation campaign in term of AUC-ROC and F-measures.

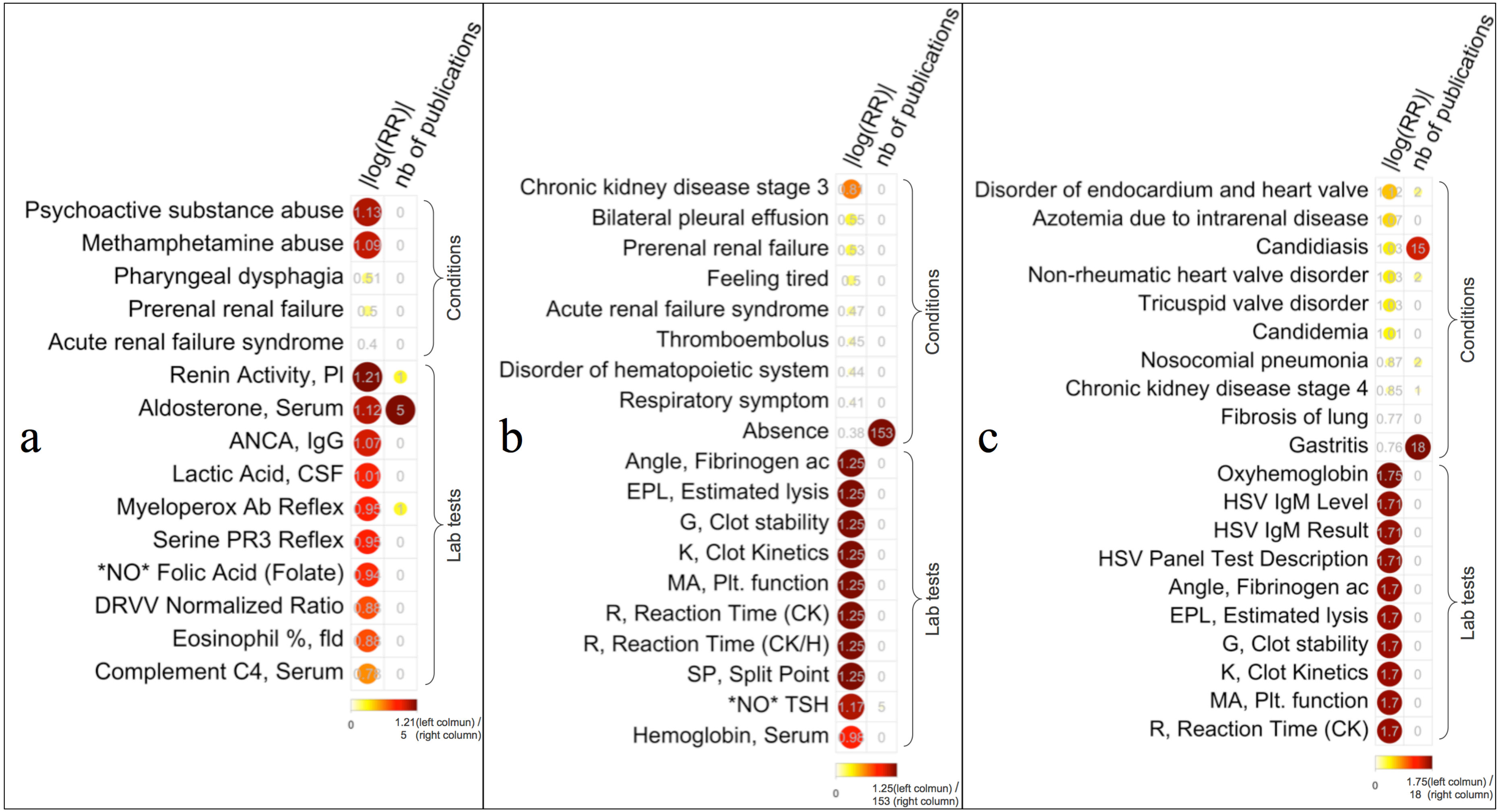

Fig S2. Details of phenotype profiles associated with dose reductions of labetalol (**a**), sildenafil (**b**) and warfarin (**c**). Features are associated with a statistically significant p-value (hypergeometric test, $p < 0.05$, Bonferroni correction for multiple testing), and ordered by the absolute value of the log of the Risk Ratio, in the first column. For interpretation purpose, the number of articles in PubMed that mention both the drug and the phenotype is also provided, in the second column.

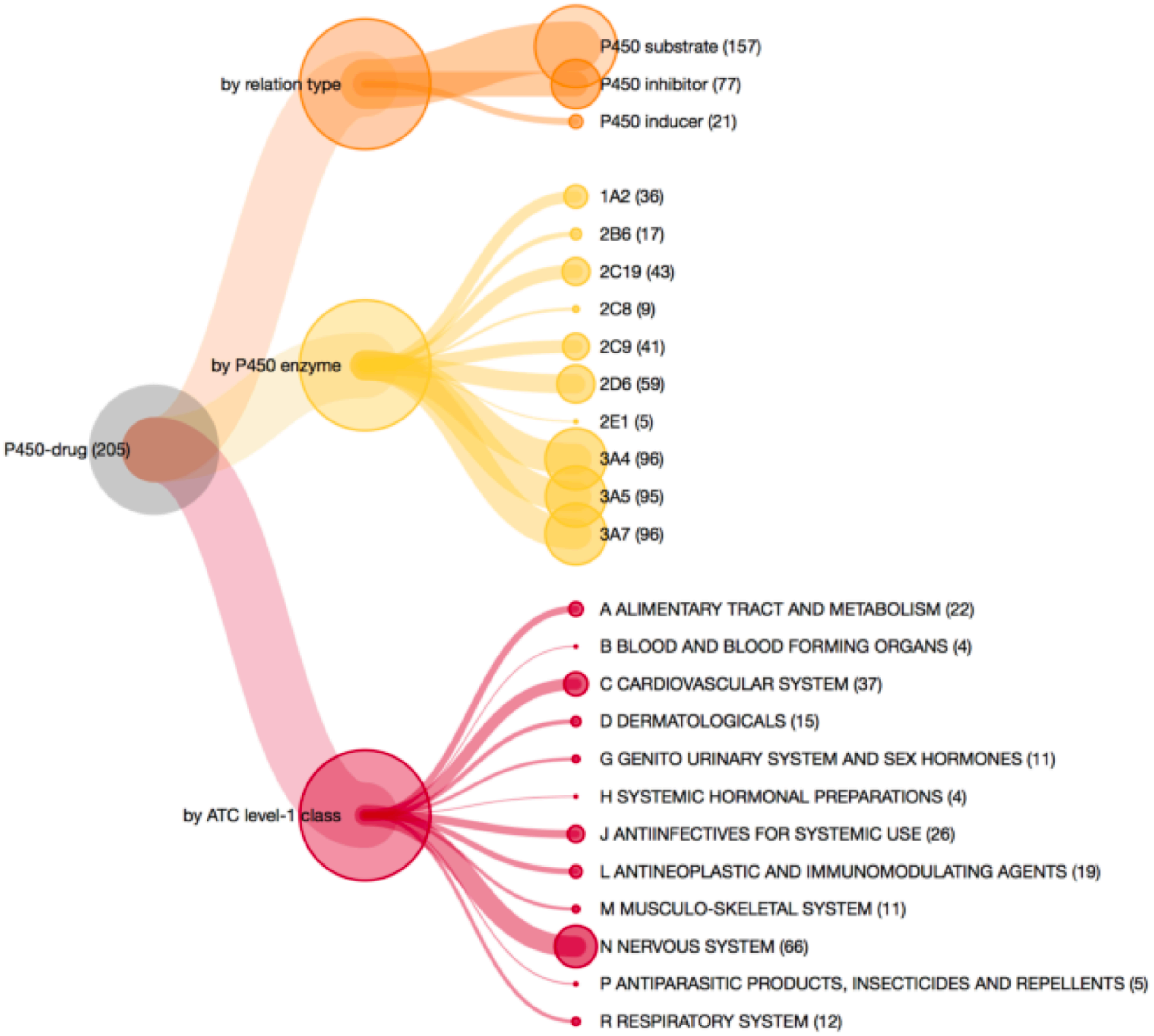

Fig S3. 25 sets of P450-drugs considered in this study, organized regarding 3 criteria: the relation type they have with P450 enzymes, the P450 enzyme they are interacting with, and the ATC class (1st level) they are part of. Accordingly, one drug may belong to several sets. The size of each node is proportional to the size of the set, provided in parenthesis.

Data File S4. List and grouping of P450-drugs considered in this work.
This JSON file lists the sets of drugs considered and the drugs they are composed of. It includes the size of each set and RXCUI of single drugs.

Supplementary methods. Details on the computation of the p-value, Risk Ratio and Information Content.

p-value computation with the hypergeometric test
In our study, a p-value quantifies how much a feature is associated with a dose reduction or increase for a single P450-drug or for a set of drugs. Smaller the p-value is, higher is the statistical significance of the features' association with the outcome under study. Computing the p-value of the feature p, for a dose reduction (or a dose increase) of the drug d necessitates four parameters: M, the population size, is here the cardinality of the set of dose reductions and continuations for the drug d (denoted dr_d and dc_d) or $M = | dr_d \cup dc_d |$; m, is the cardinality of the set of dr for the drug d, denoted $m = | dr_d ||$; N, the sample size, is the cardinality of the set of dr and dc associated with the feature p or $N = | dr^p \cup dc^p |$; and n, is the cardinality of the set of dr for the drug d associated with the feature p, denoted as $n = | dr_d^p |$. A p-value is computed for each feature associated with either a dr or a di interval.

Risk Ratio computation
The Risk Ratio (RR) measures the difference of association of a feature in two patient populations: the patients with dose reduction (or increase) of a drug; and the patient with no dose reduction (or increase) of the same drug. For a drug d and a feature p, we compute the RR using the same parameters as the hypergeometric test:

$$RR(d,p) = \frac{\frac{n}{m}}{\frac{N-n}{M-m}} \ .$$

Information Content computation
The information Content (IC) of conditions is used to avoid considering features that may be either too common or too uncommon in our set of Electronic Health Records. We computed the IC of a feature p as follows:

$$IC(p) = -\log\left(\frac{k}{K}\right)$$

where K and k depends on the kind of feature considered. For diagnostic codes, condition mentions in clinical notes and laboratory test ordering, K is respectively the total number of visits, clinical notes or laboratory test orders and k is the number of visits, clinical notes or laboratory test orders associated with p.
